# Mobile Messaging Services-Based Personal Electrocardiogram Monitoring System

**DOI:** 10.1155/2009/859232

**Published:** 2009-08-20

**Authors:** Ashraf A. Tahat

**Affiliations:** School of Electrical Engineering, Princess Sumaya University for Technology, Amman 11941, Jordan

## Abstract

A mobile monitoring system utilizing Bluetooth and mobile messaging services (MMS/SMSs) with low-cost hardware equipment is proposed. A proof of concept prototype has been developed and implemented to enable transmission of an Electrocardiogram (ECG) signal and body temperature of a patient, which can be expanded to include other vital signs. Communication between a mobile smart-phone and the ECG and temperature acquisition apparatus is implemented using the popular personal area network standard specification Bluetooth. When utilizing MMS for transmission, the mobile phone plots the received ECG signal and displays the temperature using special application software running on the client mobile phone itself, where the plot can be captured and saved as an image before transmission. Alternatively, SMS can be selected as a transmission means, where in this scenario, dedicated application software is required at the receiving device. The experimental setup can be operated for monitoring from anywhere in the globe covered by a cellular network that offers data services.

## 1. Introduction

According to a World Health Organization (WHO) estimate, cardiovascular disease kills almost seventeen million people around the globe each year [1], with around twenty million people at a risk of sudden heart failure. Some of these lives can often be saved if acute care and cardiac surgery are provided within the so-called golden hour. Therefore, patients who are at risk require that their cardiac health to be monitored frequently whether they are indoors or outdoors so that emergency treatment can be given if problems arise [2]. Telemedicine is widely considered to be part of the inevitable future of the modern practice of medicine. It is gaining more and more momentum as a new approach for patients' surveillance outside of hospitals (at home) to encourage public safety and to facilitate early diagnosis, treatment, and increased convenience. Defined as the “use of advanced telecommunication technologies to exchange health information and provide health care services across geographic, time, social, and cultural barriers,” telemedicine is currently being used by doctors, hospitals, and other healthcare providers around the world [3]. Conventional telemedicine systems using Public Switched Telephone Network (PSTN) land lines are already available to enable a doctor to monitor a patient remotely for home care or emergency applications [4, 5]. Also, the mobile phone has been recognized as a possible tool for telemedicine since it became commercially available, and in the past few years, some parties have shown that with a biosignal acquisition unit connected to a notebook computer, which interfaces to a cellular phone equipped with a built-in wireless modem, vital signs can be transmitted from an ambulance to a hospital in a store-and-forward mode [6, 7] or in real-time mode [8]. Moreover, newer cellular access technologies, such as GPRS, EDGE, 3G, and WiMAX provide for much higher data transmission speeds (rates) than the basic 2G GSM cellular system offering future telemedicine solutions endless choices for high-end designs [2]. However, these relatively new wireless technologies are deployed mostly in or around crowded high-income metropolitan areas. Therefore, the majority (80.8%) of the 3.8 billion cellular phone users in the world are still 2G GSM users [9].

In this paper, we describe a telemedicine system based on mobile messaging services, namely, Short Messaging Service (SMS), which is an integral part of the original 2G GSM cellular system and subsequent generations, and Multimedia Messaging Service (MMS), which became available as part of the 2.5G cellular technologies and onward. This system transfers a patient's Electrocardiogram (ECG) signal and body temperature and can also be expanded to include other vital signs. Our motivation to use mobile messaging services is that not only does it provide an alternative means of transmission in a cellular communication system but it is a more versatile and convenient option since all new phones are SMS and MMS capable. Additionally, under certain circumstances, as explained in Section 5, SMS can be the only means of transmission in a cellular system.

In Section 2 of this paper, we describe the architecture of the mobile telemedicine system and the underlying principles. Section 3 discusses the patient ECG signal and temperature acquisition unit. Sections 4 and 5 discuss transmission via MMS and SMS, respectively. And we conclude in Section 6.

## 2. System Concept

**Figure 1:**
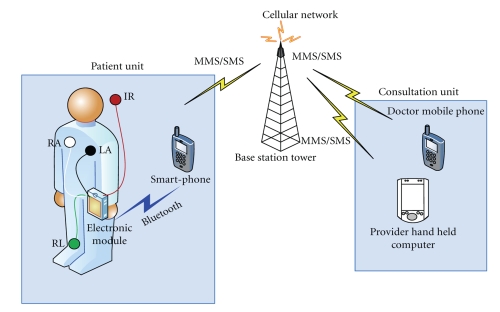
Mobile monitoring and measurement system.

The proposed mobile telemedicine system is shown in Figure 1. The patient (client) and the health-care professional can be located anywhere in the globe where there is 2G or 2.5G cellular network coverage. The patient's ECG, body temperature, and other vital-signs if desired, can be acquired by the patient himself under follow-up scenario, for example. Alternatively, the patient can be assisted by a family member or a health-care professional in more serious cases depending on particular patient's case. The primary purpose is to monitor patients cardiac activity if there is a chance that patient has cardiac problems such as an irregular heartbeat or arrhythmia that require close monitoring or that occur intermittently. The signal acquisition process is performed by attaching the ECG electrodes (three in the present setup) to the patient's body at designated places as is normally done in a typical single-lead ECG setup, and the Infrared temperature sensor is pointed to the forehead. The ECG signal and temperature acquisition unit acts as a temporary storage for the acquired ECG signal and temperature readings; then it communicates with the smart mobile phone via a Bluetooth connection, which can be established through a Bluetooth transceiver. The mobile phone in-turn is tasked with plotting the ECG signal before it is captured and saved as an image, and then sent as an MMS if desired. The other option is to send an SMS that contains the ECG samples and a temperature reading if only 2G cellular network coverage is available. Additionally, the received data on the smart-phone is written continuously in blocks of 16 kByte to the secure digital (SD) memory of the smart-phone for future retrieval and analysis, where a one-hour long single-channel ECG will occupy 900 kByte of memory space.

When the MMS or SMS message reaches their destination mobile phone (or PDA), it is displayed directly on the screen, or it can be downloaded on to a personal computer for more convenient and larger display and storage.

## 3. The Patient Unit

**Figure 2:**
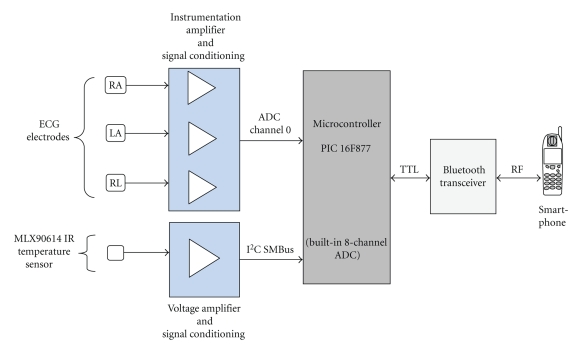
The client unit of the mobile ECG and temperature measurement system.

The patient unit is comprised of the ECG signal and temperature (vital-signs) acquisition module and a smart mobile phone. The core of the signal acquisition module is a microcontroller (PIC 16F877) as shown in Figure 2. The microcontroller acquires the amplified and conditioned signals and then performs the interface with the Bluetooth transceiver using the built-in UART.

### 3.1. ECG Leads

**Figure 3:**
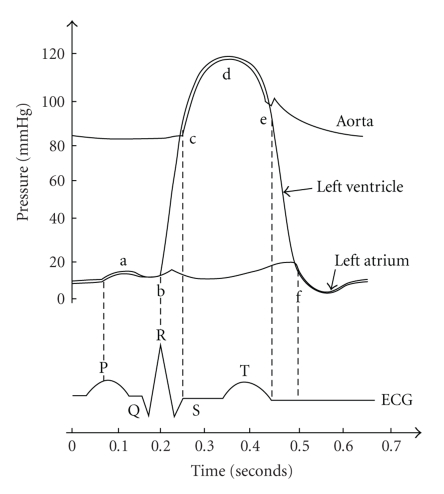
A typical ECG with relevant features.

The ECG is a graphical representation of electrical activities of the heart. The resulting heart dipole vector is used as a source for the ECG signal, which is the spatial sum in space of all distributed dipoles in cardiac tissue. A normal electrocardiogram with its characteristic patterns and significant points and intervals is shown in Figure 3.

The amplitude of a QRS-complex is typically about ±1 −2 mV. We feed the signals from the three ECG electrodes, Left Arm (LA), Right Arm (RA), and Right Leg (RL), into the inputs of the designed instrumentation amplifier conditioning circuit of an overall gain of 800. The single-channel ECG signal that is fed into the microcontroller is composed of Lead-I (i.e., LA, RA), which is the lead most often chosen for cardiac monitoring.

The signal is bandpass-filtered with a frequency range from 0.15 to 50 Hz. The ECG derived from the surface bears frequency components up to a maximum frequency of 100 Hz, but most of the spectrum is concentrated below 40 Hz.

### 3.2. Body Temperature Sensor

We used a special rapid response, low-cost, integrated, noncontact, Infrared (IR) temperature sensor IC, the MLX90614, that delivers medical accuracy over a wide operating range in our reference design. This particular temperature IC delivers ±0.1°C measurement accuracy in the object temperature range of 36°C–39°C and the wide ambient temperature range. It has on-chip amplification, signal processing, and conditioning circuit. We interfaced the temperature sensor to the PIC16F877 microcontroller using the I^2^C port on the microcontroller, which supports SMBus voltage levels.

### 3.3. The Microcontroller

**Figure 4:**
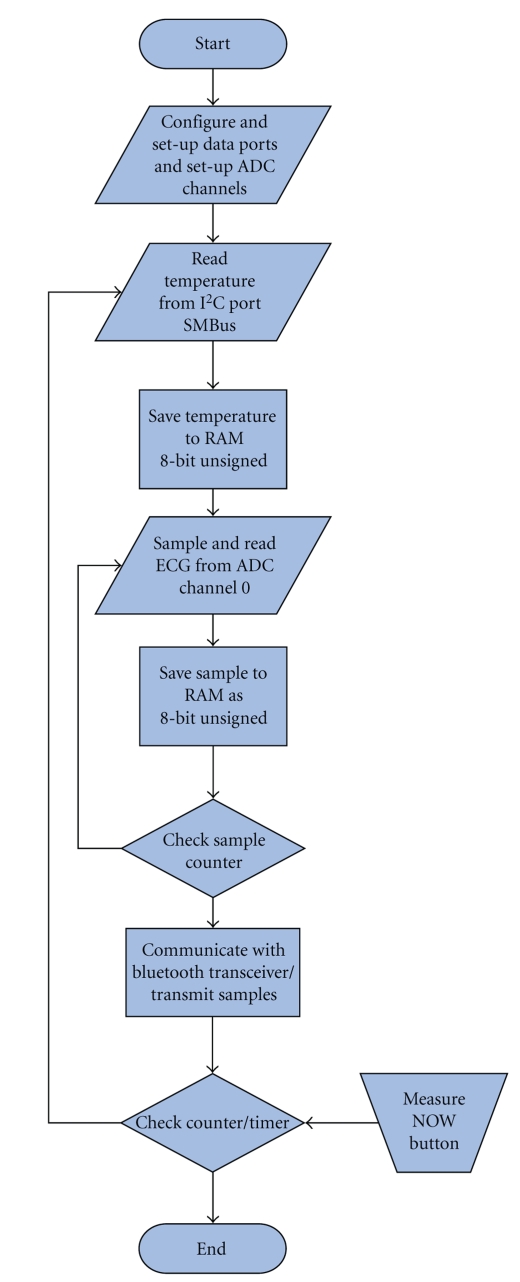
The microcontroller software flowchart.

The PIC 16F877 is an 8-bit microcontroller, which has an on-chip eight-channel 10-bit Analog-to-Digital Converter (ADC). The amplified and conditioned ECG signal is fed to channel-0 of the microcontroller. Also, upon command, the microcontroller reads the temperature sample stored in the RAM of the MLX90614 through the I^2^C port and then converts and stores it in its RAM as two 8-bit unsigned integers (0–255). The microcontroller also continuously samples the ECG signal on channel-0 with a sampling interval of 4 milliseconds (250 Hz), which is sufficient to resolve a maximum frequency component of about 125 Hz in the frequency content of the ECG signal according to the sampling theorem. We oversample to get a smoother plot of the ECG signal, but it is a modest oversampling because we do not want to overburden the microprocessor of the smart-phone while continuously plotting and refreshing the screen. Samples are written directly to the serial port in asynchronous mode using the built-in UART of the PIC16F877, which is used to communicate with the Bluetooth transceiver using TTL voltage levels. We show in Figure 4 a flowchart of the microcontroller program.

### 3.4. Bluetooth Transceiver

Bluetooth is an industrial specification for wireless Personal Area Networks (PANs) [10]. Bluetooth provides a way to connect and exchange information between devices such as mobile phones, laptops, PCs, printers, digital cameras, and video game consoles over a secure, globally unlicensed short-range radio frequency. The Bluetooth specifications are developed and licensed by the Bluetooth Special Interest Group. It is a standard communications protocol primarily designed for low power consumption, with a short range (1 meter (0 dBm), 10 meter (4 dBm), and 100 meter (20 dBm)).

We used the LinkMatik 2.0 Bluetooth transceiver module, which is a class 1 model that has an approximate range of 100 meters. The asynchronous data from the PIC16F877 microcontroller is delivered to the LinkMatik Bluetooth module on the serial port at a speed of 9600 bps. The Bluetooth module is configured as a *Master*, and the mobile phone is considered to be functioning as a *Slave.* The signal acquisition unit sends data to the Bluetooth module, which transmits data continuously, in blocks of 500 ECG samples (a duration of 2 seconds long of lead-I)) plus a one temperature reading. The data is sent as raw binary bytes.

The Bluetooth standard aims at guaranteeing reliability and robustness with acceptable security that stems from the fact that it uses Frequency-Hopped Spread Spectrum (FHSS) technique, which generated an increased demand for applying Bluetooth technology in the healthcare world [11]. Also, Bluetooth requires authentication to restrict connectivity to devices when configured in security modes. Additionally, it uses encryption to employ secret keys where only authorized users can make data intelligible again.

## 4. ECG Transmission via MMS

MMS technology has been introduced with the development of GPRS networks and handsets. GPRS allows any handset to exchange data with a network entity through a packet-designed protocol on a multiple traffic channel circuit. Then, depending on the network and handset capabilities, the throughput of data exchange can reach 64 kbps. With this technology, it is possible for a mobile phone to send more sophisticated data than SMS messages can to the network entities. MMS technology was introduced to provide GSM subscribers with the ability to send messages that are a combination of text, images or video, and sounds. MMS is an essential component of 2.5G cellular mobile phone systems and their evolution toward current 3.5G systems. Its use for many commercial and government applications is continuously increasing [12]. Moreover, since MMS messages can contain various forms of media data such as sound and video, this will give a more realistic and lively experience to the health-care provider, which will enable other diagnosis tests to be integrated into that MMS message along with the ECG and body temperature. Most importantly, since most new phones are MMS capable, there is no need for special application software to display the ECG on the mobile phone directly, which is received as bitmap/JPEG image. Additionally, even on smart-phones and PDAs that have small to medium sized screens, most devices have a zoom-in function that will enable enlargement of an ECG graph to be inspected.

**Figure 5:**
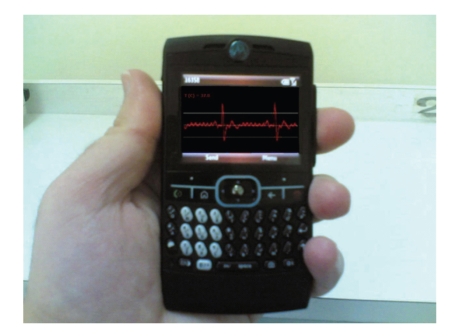
Motorola Q smart phone with the application software.

**Figure 6:**
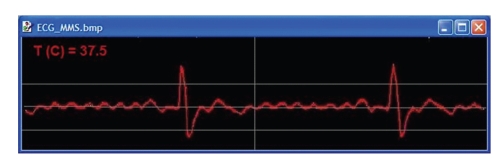
A sample image displaying ECG plot and body temperature after download to a PC.

The setup for the patient (client) unit is composed of mobile application software running on a smart-phone (or PDA) in our MMS-based telemedicine system that we have developed to transfer a patient's ECG signal and body temperature [13]. This mobile application is capable of opening a virtual communication serial port of the smart mobile phone, where in this case it is the Bluetooth driver. The software application takes the received bytes from the buffer and plots 500 bytes of ECG samples at a time (2 seconds) and displays the temperature in degrees Celsius. It also accumulates 16 kB of data samples before they are written to SD memory card of the device. The platform or Operating System (OS) used to run the application software will influence the choice of the preferred programming language used in implementing the software. The smart-phone we have used was the Motorola Q mobile phone with Windows Mobile 5 operating system, which has 64 MB RAM and 64 MB of mini-SD . The application software was developed using the C# programming language on Microsoft Visual Studio in conjunction with the Windows Mobile 5 software development kit (SDK). Figure 5 shows the Motorola Q smart-phone with the application software running and displaying the received 500 byte ECG signal and temperature via Bluetooth. The application software has a capture button, which allows for saving the display as an image for storage and to be sent as an MMS message when desired. The wave form is plotted in window with an area of 320 × 130 pixels. The amplitude resolution is 0.0144 mV/pixel for the single-lead ECG display, and the temporal resolution is 0.00625 s/pixel. Also, stored ECG plots and raw data samples can be downloaded to a PC for further processing or storage. Figure 6 shows a sample ECG plot and displayed body temperature image, which was downloaded using Bluetooth on to a Windows XP-based personal computer. The temperature is presented in three digits with a decimal point.

## 5. ECG Transmission via SMS

As we have pointed out in the introduction, newer wireless access technologies that offer dedicated data services beyond the basic 2G cellular system are deployed mostly in or around crowded high-income metropolitan areas. However, SMS is an integral part of the original 2G GSM system and its evolution. Not only does SMS offer an alternative means of transmission in a 2G cellular communication system like GSM but also at times it can be the only available or most efficient option. This can be evident when considering the vulnerable nature of the traditional wireless voice channel used by a cellular phone to establish a connection with the serving base station, including dropped calls and service denials during peak hours. SMS uses different sets of channels that are more robust than those assigned for voice (or wireless modems), which can enable a user to send and receive SMS messages at times when s/he cannot get access to the network for voice calls. This is because SMS uses control channels of a cellular system, which are used for the initial call setup rather than the regular voice traffic channels. Furthermore, SMS is being used for many commercial and government transactions and services, and this use is rapidly expanding [14].

Our SMS-based transmission scheme of the ECG signal and body temperature in the proposed telemedicine system uses the same hardware components and application software on the client smart mobile phone as those of the MMS-based one but requires additional application software to be installed on the consultation (doctor) unit at the receiving device. Also, an alternate simpler arrangement where the application software installed on the patient unit can be eliminated was demonstrated in [15], but the microcontroller program had to be augmented to perform SMS message construction and transmission. Here, as was done in the MMS transmission scenario, and in identical steps, the software application takes the received bytes from the buffer and plots 500 bytes of ECG samples at a time and displays the temperature in degrees Celsius. However, when the SMS option is selected from the *menu* button to activate transmission via SMS, we derive an ECG signal sampled with an interval of 8 milliseconds (125 Hz). This is done by taking alternate samples from the temporarily stored ECG signal which was sampled with intervals of 4 milliseconds (250 Hz). The new samples are stored in another buffer in memory, along with the temperature reading, for further processing. According to the sampling theorem, this sampling frequency is sufficient to resolve a maximum frequency of about 62 Hz in the ECG spectrum. We use this particular lower frequency for reasons that will be discussed subsequently when demonstrating SMS message structure and capacity. After completion of signals acquisition, the application software feeds the data bytes of the desired length to the SMS message construction application program interface (API), which will prompt the user to enter the consultation/server unit mobile number.

### 5.1. SMS and PDU Mode

There are two ways of sending and receiving SMS messages: by Text mode and by Protocol Description Unit (PDU) mode [16]. The text mode is just an encoding of the bit stream represented by the PDU mode. The PDU mode offers to send binary information in 7 bit, 8 bit, and 16 bit format. When using the default 7-bit characters, the SMS message, as specified by the ETSI organization documents GSM 03.40 and GSM 03.38, can be up to 160 characters long, while when using 8-bit characters, messages can contain a maximum of 140 characters. Messages that use 8-bit characters are not usually viewable by the phones as text messages; instead they are used for data in smart messaging. Unicode (UCS2) text messages use 16-bit characters, and the maximum length of the message is reduced to 70 characters only.

PDU mode can be viewed as a management mode of SMS data elements. It directs constructing the entity of the message when sending an SMS and unconstructing SMS messages when receiving them to extract the contents under the operating system environment. In PDU mode, we are able to specify the desired destination mobile number, encoding of the message content, and length of time an SMS message stored in the network if the mobile device is turned-off, and delivery receipts can be requested [16]. Also, a received SMS message can display several specifications like the sender phone number, time and date, and type of content to undertake the appropriate decoding process.

At the client unit, the API packs the temperature and ECG samples in a predetermined number of SMS messages that depends on the desired length of duration of the patient's ECG. Since we have originally sampled our ECG signal using the microcontroller ADC with 8-bit resolution and subsequently stored as 8-bit unsigned integers (bytes) in the API buffer, we use 8-bit data encoding when constructing the SMS messages. As was previously mentioned, this will allow us to load 140 samples (bytes) in each SMS message. And with a sampling frequency of 125 Hz (8 milliseconds intervals), each SMS message is capable of displaying an ECG segment of 1.12 seconds. However, we only transmit 130 samples of ECG signal and one sample (three bytes) of the temperature in each SMS message; this will leave seven unused bytes for other vital signs if desired. Although, in principle, the GSM standard allows for 255 (2^8^ − 1) messages to be concatenated and received as one long SMS message, the maximum limit set by most GSM networks allows SMS concatenation up to six messages. In our case, this will allow us to transmit up to 6.24 seconds of ECG at a time.

### 5.2. AT Commands

The AT commands are standard control tools based on GSM (07.07) to establish communication with the mobile GSM phone or modem [16]. The command sets consist of strings, which will enable the exchange of serial data, according to certain syntax rules, between the mobile and the laptop or PC at the consultation/server unit if this arrangement is preferred. As an example, “AT + CMGS = 140” is a command for sending an SMS message, where “AT” is a prefix used for all commands, “CMGS” is a description to the kind of task to be performed, and “140” is the message length.

Similarly, when a message is received by the laptop according to the AT command “AT+CMGR=1”, the program will be able to divide the message contents in order to extract the binary 8-bit samples to display the temperature and plot the ECG segment in the User Data (UD) part of the SMS(s).

### 5.3. Consultation Unit

The simplest setup for a consultation (doctor) unit would be a mobile application running on a PDA or a smart-phone. This mobile application is capable of opening the SMS messages that carry in their payload the temperature and ECG samples, decode the SMS messages, and extract the UD part to display the temperature and plot the ECG of the patient on the screen of the PDA itself. An alternate arrangement for the server unit is to establish a serial connection between the mobile phone or modem and a laptop via a serial COM port using a Bluetooth transceiver or by a direct physical connection through a standard RS232 or USB cable.

The software application running on the laptop performs the background communications with the mobile phone using AT Commands. We have chosen the latter arrangement of the consultation unit for convenience and easier interface to other Windows PC-based analysis software. The platform used to run the software will influence the choice of the preferred programming language used in implementing the software. We used Visual Basic programming language to implement the server application software for a Windows-based laptop. The software starts with initializing and registering the desired COM port.

**Figure 7:**
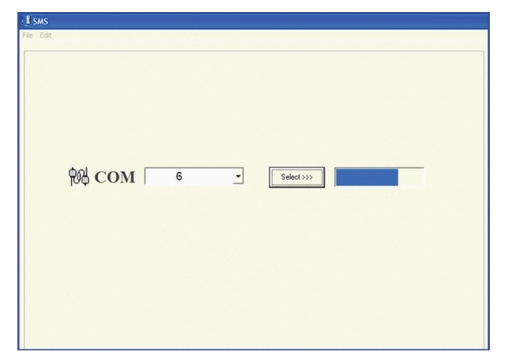
Serial port selection and registration in the application software.

As shown in Figure 7, the software main menu will be disabled until COM port number selection is correct, and connection with the mobile is made. Reading the SMS messages from the mobile phone and decoding their contents is the heart to this software. The software decodes each SMS message and extracts the time and date, originating mobile number, the transmitted patient's temperature, and ECG samples in the payload.

**Figure 8:**
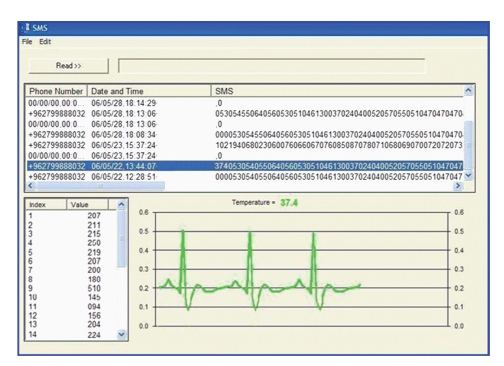
Application software interface of the receiving PC running windows.

It will also perform concatenation of received SMS messages when this option is used. The contents of the messages are displayed in the upper-half of the interface screen as shown in Figure 8. The first three digits represent temperature with an implied decimal point after the first digit from the right.

When a particular SMS message is selected from the list of received messages in the upper-half of the interface window, the software converts the ECG data points carried in the message to mille-volts, where they are listed in a table in the lower-left corner of the interface window and are also plotted to scale to the right in the designated area as shown in Figure 8. Simultaneously, the temperature value is displayed in degrees Celsius at the top of the ECG plot.

## 6. Conclusion

A proposed low-cost mobile patient monitoring system that utilizes mobile messaging services (MMS/SMSs) was designed, developed, and tested. An Infrared temperature sensor was integrated with a three-electrode ECG signal acquisition circuitry in a module that communicates with a mobile smart-phone via Bluetooth. Also, application software running on the smart-phone was developed to receive and plot the ECG signal and display the body temperature before transmission via MMS or storage in a secure digital memory card. Alternatively, SMS can be used for transmission, and a PC-based application software was developed to display the received biosignals at the consultation (doctor) unit. The new system has a significantly reduced size and weight, which improves its versatility. In addition, MMS makes it a universally capable telemedicine system with a great potential for expansion. Furthermore, SMS can be the most suitable, if not the only, method of data transmission in emergency situations in a remote area where broadband data communications (like GPRS, EDGE,…, etc.) are not available.
